# Harnessing Crop Wild Diversity for Climate Change Adaptation

**DOI:** 10.3390/genes12050783

**Published:** 2021-05-20

**Authors:** Andrés J. Cortés, Felipe López-Hernández

**Affiliations:** 1 Corporación Colombiana de Investigación Agropecuaria AGROSAVIA, C.I. La Selva, Km 7 Vía Rionegro, Las Palmas, Rionegro 054048, Colombia; llopez@agrosavia.co; 2 Departamento de Ciencias Forestales, Facultad de Ciencias Agrarias, Universidad Nacional de Colombia, Sede Medellín, Medellín 050034, Colombia

**Keywords:** abiotic stress tolerance, genebanks, germplasm collections, *ex situ* conservation, genetic adaptation, genome-wide selection scans (GWSS), genome–environment associations (GEA), genomic prediction (GP), machine learning (ML)

## Abstract

Warming and drought are reducing global crop production with a potential to substantially worsen global malnutrition. As with the green revolution in the last century, plant genetics may offer concrete opportunities to increase yield and crop adaptability. However, the rate at which the threat is happening requires powering new strategies in order to meet the global food demand. In this review, we highlight major recent ‘big data’ developments from both empirical and theoretical genomics that may speed up the identification, conservation, and breeding of exotic and elite crop varieties with the potential to feed humans. We first emphasize the major bottlenecks to capture and utilize novel sources of variation in abiotic stress (i.e., heat and drought) tolerance. We argue that adaptation of crop wild relatives to dry environments could be informative on how plant phenotypes may react to a drier climate because natural selection has already tested more options than humans ever will. Because isolated pockets of cryptic diversity may still persist in remote semi-arid regions, we encourage new habitat-based population-guided collections for genebanks. We continue discussing how to systematically study abiotic stress tolerance in these crop collections of wild and landraces using geo-referencing and extensive environmental data. By uncovering the genes that underlie the tolerance adaptive trait, natural variation has the potential to be introgressed into elite cultivars. However, unlocking adaptive genetic variation hidden in related wild species and early landraces remains a major challenge for complex traits that, as abiotic stress tolerance, are polygenic (i.e., regulated by many low-effect genes). Therefore, we finish prospecting modern analytical approaches that will serve to overcome this issue. Concretely, genomic prediction, machine learning, and multi-trait gene editing, all offer innovative alternatives to speed up more accurate pre- and breeding efforts toward the increase in crop adaptability and yield, while matching future global food demands in the face of increased heat and drought. In order for these ‘big data’ approaches to succeed, we advocate for a trans-disciplinary approach with open-source data and long-term funding. The recent developments and perspectives discussed throughout this review ultimately aim to contribute to increased crop adaptability and yield in the face of heat waves and drought events.

## 1. Introduction—The Imminence of the Threat

How plants and crops will respond to a warmer and drier climate is currently one of the most discussed multi-disciplinary questions in the fields of environmental science, ecology, and evolution. It is estimated that climate change effects may limit global crop production by at least 10% in 2050 [1,2], especially in vulnerable regions around the globe where heat, drought, and malnutrition are already substantial. Given that current agricultural resources might not be sufficient to meet future food demand [3], crop wild relatives and landraces historically adapted to dry and semi-arid environments are key sources of yet unexplored diversity with respect to major food crops [4]. Exotics may donate necessary genetic variation to make heat and drought-tolerant cultivars, or may even stand as novel crops by themselves [5,6,7] (e.g., *Lupinus mutabilis* [8]).

**Figure 1:**
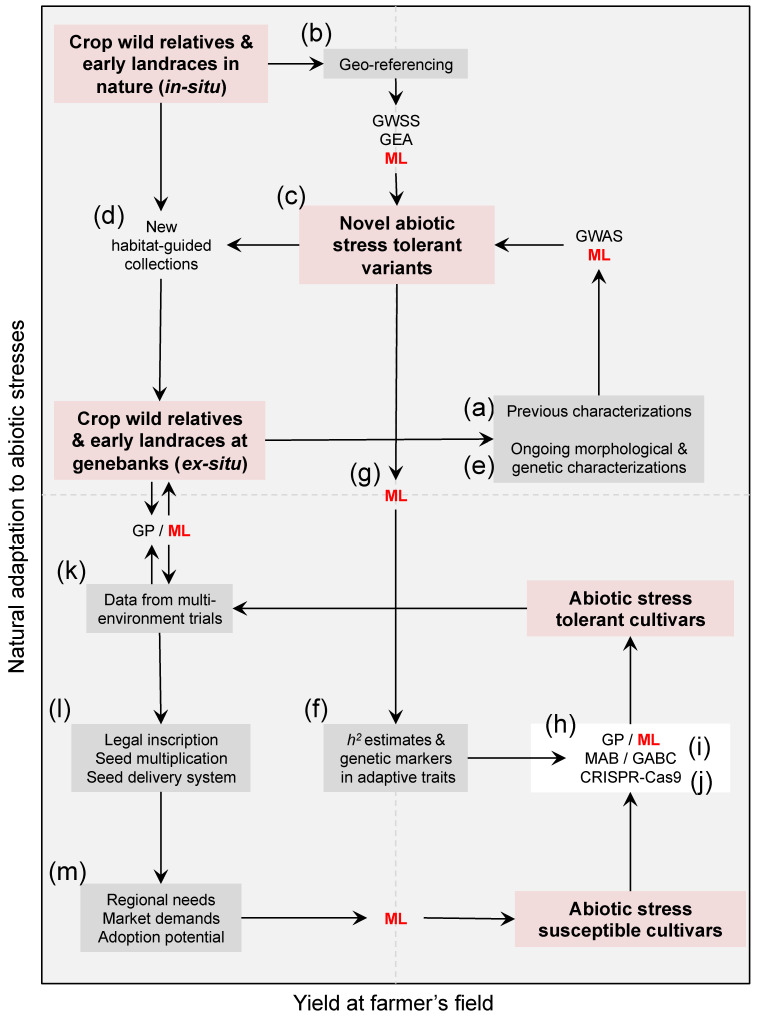
A roadmap of trans-disciplinary approaches aiming at harnessing genebank utilization for climate change research in the face of heat, and water scarcity. Compiling (**a**) previous characterizations and (**b**) geo-referencing-derived climate data/indices of available genetic resources in genebanks is a starting point to (**c**) assess the extent of abiotic stress tolerance among existing accessions, and the need of (**d**) new habitat-based population-guided collections targeting isolated pockets of cryptic diversity in dry and semi-arid regions. Planning question-oriented collecting trips of crop wild relatives and hidden landraces across contrasting environments/agro-ecologies is needed now more than ever, despite a century of gathering and preserving diversity in plants throughout genebanks. Coupling *ex situ* agro-ecological screenings together with (**e**) ongoing *in situ* genebanks characterizations for morphological and genetic variation is essential to define (**c**) putative tolerant reference collections, while understanding the (**f**) heritability (*h^2^*) of adaptive traits and their genetic architecture (i.e., underlying genes) via genome-wide selection scans (GWSS), genome–environment associations (GEA), and genome-wide association studies (GWAS). Since identifying these novel sources of heat and drought tolerance demands merging heterogeneous datasets, (**g**) machine learning (ML, in red letters) promises speeding up genebank characterization. The distinction that clustering (Table 1) and ML (Figure 2 and Table 2) strategies can provide between abiotic stress tolerant and susceptible accessions is essential to (**h**) transfer useful genetic variation from wild crop donors and early landraces into elite cultivated lines, either by designing (**i**) genomic-assisted breeding programs such as genomic prediction (GP) and inter-specific marker- and genomic-assisted backcrossing (MAB and GABC) schemes, or by envisioning (**j**) multi-trait gene editing strategies (e.g., CRISPR-Cas9). Once (**k**) abiotic stress tolerant varieties are validated across different environments, (**l**) legal inscription, seed multiplication, seed delivery system to farmers’ associations, and (**m**) follow-up given the regional needs, market demands, and adoption potential, are necessary downstream validation steps. These heterogeneous datasets are also likely to be inputted into ML, and in turn feedback new needs beyond heat and drought tolerance such as other types of resistances and nutritional quality. For ML to succeed speeding up the breeding of heat and drought-tolerant crops, there must be long-term funding to generate and maintain an assortment of datasets at each step, which in turn need to be publicly available through open access repositories from various geographic locations. Red boxes highlight different reservoirs of wild and cultivated diversity within the Cartesian space, gray boxes are mixed datasets built around these collections, and connectors are methodological approaches.

However, efforts to capture and pyramid target tolerant variants from the wild still face major challenges. First, identifying useful variation in wild accessions through field trials (Figure 1a) has been inefficient because of the disparity in growth rates and phenologies [9]. This limitation is reinforced by a complex inheritance of the abiotic stress tolerant phenotypes involving many genes of low effects and several environmental interactions [10]. Second, the domestication syndrome typically neglected heat and drought tolerance in the majority of crop species [11,12] because these adaptations in wild populations (Figure 1b) tend to perpetuate vegetative phases, delay reproductive stages, and therefore compromise overall yield [13]. Due to this, transferring variation from wild exotic donors into elite lines may induce undesired linked trait variation such as flowering delay and reduced crop yields (e.g., in *Phaseolus* species [14]).

With the advent of the genomic era, heat and drought tolerance molecular pathways have been extensively studied [15,16,17], and numerous candidate genes and genetic marker associations have already been identified and validated (e.g., ABA-dependent or independent pathways, and *ASR*, *DREB*, and ERECTA-encoding genes [18,19,20,21]). Coupling these comprehensive datasets with novel analytical tools harbors the potential to identify and unlock useful genetic variation among crop wild relatives and landraces to challenge with abiotic stresses. In the following sections, we outline the main avenues to establish novel sources of abiotic stress tolerance variation from wild crop relatives and semi-domesticated landraces (Figure 1c), and discuss modern genomic-assisted strategies to utilize these variants to obtain heat and drought-tolerant elite crop lines. We finish by emphasizing the need to enable the construction and long-term maintenance of big heterogeneous dataset repositories capable of powering these innovative predictive strategies.

## 2. Valuing the Wild—Strategies to Identify Naturally Available Exotic Variants

Domestication has been the most ambitious evolutionary experiment humanity has ever embarked on [11,22]. Being the longest running selection trial [23], it has served as a playground for geneticists and biologists to explore the patterns and processes during crop evolution [24,25,26]. For instance, it has allowed humans to test hypothesis such as whether dual domestication syndromes have recruited the same genetic variants in parallel [27], or whether genomic divergence is more prone to harbor signatures of selection due to reduced recombination and increased drift [28]. In this way, domestication has invited researchers to study the repeatability of evolution [29], and the relative role of isolation, migration, and hybridization [30], long standing questions in evolutionary biology [31,32,33]. Similarly, studying crop evolution has proven enlightening, and addressing their wild relatives’ natural adaptation to distinct habitats (Figure 1b) informs on how plant phenotypes may react to a changing climate [34]. After all, natural selection has already tested more options than humans ever will [35]. 

### 2.1. On the Necessity of Broadening the Germplasm

Natural selection rarely misses key adaptive improvements. For instance, it has already improved the efficiency of photosynthesis and water use [35], which are major developments to enhance the yield potential that has increased little in recent decades. Therefore, inferring *in situ* genetic adaptation to heat and drought stresses, usually from *ex situ* geo-referenced widespread collections of crop wild relatives, has become a prerequisite to capture these naturally available exotic variants. Unfortunately, after a century of collecting and preserving diversity in plants throughout germplasm banks, living seed collections that serve as repositories of genetic and ecological variation [36], *ex situ* collections do not necessarily fully span the ecological niche of agricultural crop relatives [37,38] as to establish new sources of genes for improving complex adaptive traits. Nowadays, wild relatives are still discovered [39], and further expeditions (Figure 1d) are needed (e.g., in the ecological hyper-variable and species-rich neotropics) [40,41]. Because isolated pockets of cryptic diversity still persist, novel habitat-based population-guided collections for genebanks are paramount, now more than ever.

### 2.2. Going Global by Adapting Local

Germplasm collections should be used not only to introduce exotic variation but also to avoid genetic erosion and increase long-term adaptability to climate change by making crops more resistant to abiotic stresses such as heat and drought. Local adaptation to abiotic factors can be studied using historical climate at the habitats where geo-referenced germplasm accessions were originally collected (Figure 1b). If an ecological balance [42] between genotypes and environments can be assumed [43,44] (Humboldt’s ‘harmony in nature’), geo-referencing and repositories of *in situ* climate variables can then be used to compute adaptive capacity. The former condition typically applies for landraces and crop wild relatives because they have occupied local niches long enough as to be shaped by natural selection forces [45]. This approach has typically relied on clustering algorithms to predict (Table 1) not only drought tolerance, water use efficiency [46,47], and thermal tolerance [48], but also resistance to pathogens [49], and aluminum toxicity [50] in a wide range of landraces and wild species.

Genomic analytical tools (Figure 1e) commonly coupled with environmental variables in order to reconstruct the genetic architecture (Figure 1f) of adaptive trait variation to abiotic stresses are genome-wide selection scans (GWSS) [51] and genome–environment associations (GEA) [52]. Both approaches aim to capture the signatures of selection to different environments by retrieving those genomic regions that segregate and are fixed among contrasting habitats (e.g., arid vs. wet regions) [53,54]. The strategies differ in that the former uses outlier tests given a background (‘baseline’) distribution, usually within a Bayesian framework [55], while the latter relies on mixed linear models (MLMs) that explicitly incorporate covariates as random effects [56]. Because these analyses may be misleading [57,58], if confusing factors [33,59] are not appropriately accounted for, MLMs are nowadays the preferred method to describe the genetic basis of local adaptation in germplasm collections [60]. Other major improvements are the use of indices, rather than raw environmental variables, that summarize precise physiological processes (e.g., thermal thresholds and potential evapotranspiration models to infer heat/drought stress) [10,18,19,20], and the collection of spatial high-resolution climate data to make accurate predictions at the regional [61] and micro-habitat [62,63] levels.

## 3. On the Need to Speed-Up

By studying local adaptation in crop-related wild collections and landraces using available phenotypic data (Figure 1a,e) and geo-referencing (Figure 1b), and uncovering the genes that underlie complex adaptive traits (such as root morphotypes, phenology and fruit/seed set, Figure 1f), natural variation has the potential to meet the global food security demands of a drier world [4]. However, unlocking adaptive genetic variation hidden in early landraces and related wild species (Figure 1c) remains a major challenge. The rate of genetic gain for abiotic stress tolerance in elite lines via traditional backcrossing schemes [64,65,66] may be limited due to high phylogenetic distance [67,68] and low hybridization levels. To overcome the bottleneck of pyramiding allelic variants from the wild into elite lines (Figure 1h), marker-assisted backcrossing (MAB) [69,70,71,72,73], predictive breeding [74], and gene editing [75] are promising alternatives.

### 3.1. Going Quicker by Selecting (Poly) Genomically

MAB recurrent selection strategies (Figure 1i) have proven to be useful for the introgression, from exotic germplasm, of simple Mendelian traits, which are those regulated by few genes such as resistance to biotic threats (e.g., [73]). However, by definition, MAB may be unable to trace quantitative traits such as heat and drought tolerance that are typically polygenic—regulated by many low-effect genes [76]. A last-generation analytical innovation that arose by merging quantitative genetics theory with genomics is predictive breeding, more commonly known as genomic prediction (GP) [74,77,78].

**Table 1:** Non-parametric and parametric classification approaches that can assist clustering efforts to differentiate between abiotic stress tolerant and susceptible germplasm accessions. Habitat types and local adaptation to heat and drought stresses can be inferred using climate variables and physiological indices from each accession’s geo-referencing (Figure 1b) because crop wild relatives and landraces have occupied local niches (e.g., arid vs. wet regions) long enough as to be shaped by natural selection. Predicted thermal tolerance and water use efficiency, together with other data types (Figure 1e,f), can then be merged (Figure 1g) in order to identify and unlock novel sources of heat and drought tolerance. The trained classification may also speed-up the utilization of these tolerant variants by genomic-assisted breeding techniques (Figure 1i). ML approaches (Table 2) are also capable of including further data types for more cohesive multi-dimensional predictions (e.g., Figure 1m).

Approach	Method	Description of the Method	R Package/Tool	Method’s Reference	Example (Accessions × Markers)
**Non-Parametric (Reduction-Based)**	K-means	Each observation belongs to the cluster with the nearest mean. It minimizes the distance between points labeled to be in a cluster and a point designated as the center (mean)	*Stats* & *optCluster*	[79,80]	Maize—2022 × 65,995 [81]
					Ryegrass—1757 × 1,005,590 [82]
	Partitioning Around Medoids (PAM)	It minimizes the distance between points labeled to be in a cluster and a point designated as the center (medoid) of that cluster. PAM chooses data points as centers (medoid) and works with a generalization of the Manhattan Norm to define data points distance	*cluster* & *optCluster*	[83]	Maize—260 × 11,296,689 [84]
	Clustering large applications (CLARA)	It extracts multiple sample sets from the dataset and uses the best cluster as output. It uses PAM for each sample	*cluster* & *optCluster*	[83]	90 × 5000 [85]
**Non-Parametric (Distance-Based)**	Hierarchical clustering (Hclust)	It is a method of cluster analysis that seeks to build a hierarchy of clusters	*hclust* & *optCluster*	[86]	Barley—1816 × 1416 & Wheat—478 × 219 [87]
					Oat—131 × 3567 [88]
	DIANA (Divisive analysis)	It first places all objects in a cluster and then subdivides them into smaller clusters until the desired number of clusters is obtained	*cluster* & *optCluster*	[83]	These algorithms were systematically compared, and included K-means, PAM, CLARA, Hclust, DIANA, and AGNES [89]
	Agglomerative Nesting (AGNES)	It initially takes each object as a cluster, afterwards the clusters are merged step by step according to certain criteria, using a single-link method	*cluster* & *optCluster*	[83,90]	
	AWclust	The first step of AWclust is to construct the ASD matrix between all pairs of individuals in the sample. The second step is to apply hierarchical clustering to infer clusters of individuals from the ASD matrix using Ward’s minimum variance algorithm	*AWclust*	[91]	Olive—94 × 8088 [92]
					Pepper—222 × 32,950 [93]
**Parametric**	TESS3	Geography is one of the most important determinants of genetic variation in natural populations. Using genotypic and geographic data, *tess3r* provides estimates of landscape population genetic structure	*tess3r*	[94]	These algorithms have been widely used and compared among them [95], and with other non-parametric methods [96]
	fast STRUCTURE	STRUCTURE uses the core Bayesian principle of comparing likelihoods. Prior information about study samples can be supplied to further shape the unsupervised clustering	*fast STRUCTURE*	[97]	

GP uses historical phenotypic data [98] to calibrate marker-based infinitesimal additive predictive models [99,100,101], which provide a more comprehensive representation of a quantitative polygenic trait than traditional genetic mapping [102]. Hence, GP offers a key path to assist the introgression breeding of abiotic stress tolerance from the wild. GP’s predictive ability can be significantly enhanced after performing *a priori* weighted trait mapping through more conventional methods such as quantitative-trait loci (QTL) mapping [103], or genome-wide association studies (GWAS) [104], even across SNP-genotyped multi-parental populations [105,106,107,108,109]. GP may also go beyond breeding efforts, and feed back on optimizing restoration [102] and genebank characterization [110] (e.g., by predicting seed morphometric traits [111] and yield [112,113]) of thousands of germplasm accessions that could hardly be tested in field trials.

### 3.2. Fast Editing

MAB is likely to mutate into genomic-assisted parental selection (GAPS) [114] and Backcrossing (GABC) [115] as the preferred strategies for the introgression breeding (Figure 1i) of abiotic stress tolerant genetic alleles from crop wild relatives that have not undergone the domestication syndrome. Even though these scopes tend to underrate GWAS, fine-mapping is still needed for future usage of more target gene editing strategies (Figure 1j) such as CRISPR-Cas9 [75], for which a precise location of (few) major target genes is mandatory. This technique allows editing or disrupting target genes and regulator elements with a proven effect on the phenotypes. It originates from a natural system that provides bacteria with adaptive immunity to virus [75] that is co-opted to shuffle genetic variants already present in nature. Because of this, CRISPR-Cas9 does not strictly lead to genetically modified organisms (GMOs) *s.s*. As a proof of concept, the feasibility of this technology for drought tolerance has been validated in model plant species such as *Arabidopsis* [116], and crops with consolidated tissue culture protocols like tomato [117,118]. It has even demonstrated its potential for multi-trait quantitative editing [119] in orphan crops like groundcherry (*Physalis pruinosa)* [120]. However, its applicability in species with recalcitrant tissue culture is still limited, and most plants with enhanced tolerance phenotypes have displayed a trade-off with reduced crop yield [12]. A promising approach to uncouple abiotic stress tolerance from plant growth is to edit tissue-specific pathways for target drought traits [121] that could be improved to obtain heat and drought tolerant varieties more efficiently [122].

Furthermore, predictive breeding and gene editing should not be conceived as separate, non-overlapping approaches. Integration of molecular genetics and genomic analyses with more quantitative approaches has the potential to speed up various other efforts in addition to understand how plants are naturally adapted to drier climates, aiding in the innovation of biotechnological strategies and breeding programs to generate elite lines that are more productive and more capable of adapting to anticipated heat waves and drought events [123]. This is particularly true for complex traits, because even though CRISPR-Cas9 can quickly transfer major elements responsible for the abiotic stress tolerance response, its omni/polygenic nature demands assistance from other approaches like GP. Merging molecular mechanistic details that are often overlooked by genome-based assisted selection, with GP, breeding simulations [99,124], and speed breeding [125,126], would confer a major opportunity for robust whole-genome forecasting of complex and polygenic drought/heat tolerance phenotypes [124].

## 4. A Way Forward via Machine Learning

Despite the undertaken GWSS and GEA approaches having demonstrated their utility in identifying naturally available genetic adaptation to heat and drought (Figure 1c), these predictions wait to be contrasted in more systematic ways with GWAS results, GP estimates (Figure 1i), and field trials (Figure 1k), a target task for a trans-disciplinary multi-dimensional synthetic approach such as machine learning (ML).

**Figure 2:**
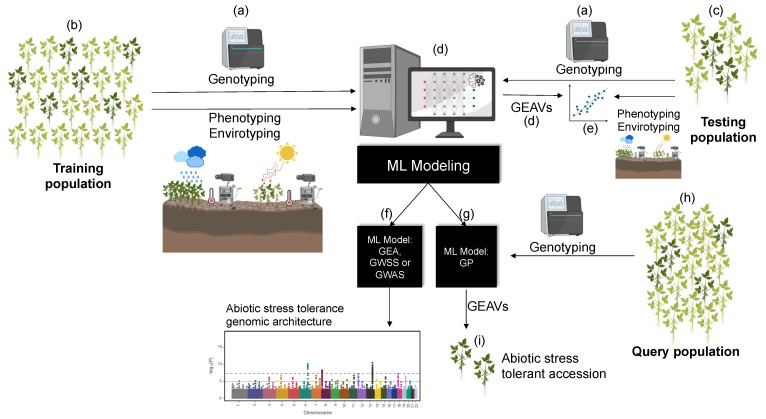
A pipeline for machine learning (ML) applications capable of predicting abiotic stress tolerant and susceptible germplasm accessions. First, a subset of the germplasm collection is (**a**) characterizing genomically, phenotypically (whenever possible), and environmentally (i.e., abiotic stress adaptation indices based on geo-referencing). This subset is later on partitioned between (**b**) training and (**c**) testing populations. The training population is used to calibrate (**d**) ML models that aim using genomic information to predict genomic estimated adaptive values (GEAVs, an analogous rank to the polygenic risk score (PGS) and genomic estimated breeding value (GEBV) from the quantitative genomics literature, e.g., [102,136]). The computer screen depicts a hypothetical hidden neural network (HNN) algorithm, which is one among many potential ML tools; the repertoire includes several regressions, classification, and deep learning models, thoughtfully reviewed this year by Sebestyén et al. [137] and Tong and Nikoloski [138]. Meanwhile, the testing population is used to compute the (**e**) unbiased predictive ability of the model by comparing the GEAVs with the recorded environmental (or phenotypic) abiotic stress tolerant/susceptible indices. Broadly speaking, calibrated and validated ML models can serve two main purposes when applied on germplasm collections. First, (**f**) they could enhance our knowledge on the genomic architecture (i.e., genetic basis) of abiotic stress tolerance via ML-based genome-wide association studies (GWAS), and on the genomic landscape of adaptation via ML-based genome-wide selection scans (GWSS) and genome–environment associations (GEA). Second, (**g**) calibrated and validated ML models can be applied on a (**h**) query population such as extended germplasm samples for which environmental-based indices or phenotyping are not viable, informing GEAVs and (**i**) abiotic stress tolerance on a wider genepool. Clusters of abiotic stress tolerance and susceptibility based on phenotypic information and/or environmental-based indices can be built using traditional classification tools such as the ones listed in Table 1, or may also leverage ML prediction approaches (Table 2).

As GP, ML output focuses on optimizing the predictive ability, or ‘recall’ rate, of a given classification (e.g., tolerant vs. susceptible accessions) (Figure 2 and Table 2), usually relying on a ‘supervised’ training with ‘labeled’ data (e.g., accessions for which drought/heat [127] tolerance are already measured or environmentally-estimated, Table 2). An advantage of ML is that it deals more efficiently with high-dimensional inputs of heterogeneous variables (Figure 1g) (‘features’) without *a priori* knowledge of their joint probability distribution [128], bypassing the ‘curse of dimensionality’ and a complex systems thinking (Figure 1l,m).

ML has long ago been used for ecological niche modeling [129,130] and functional genomics [131]. However, ML has started permeating, until very recently, other approaches more relevant to this review such as GWSS [128,132] and GP [133,134,135]. In this latter example, ML techniques (i.e., deep learning) outperformed GP’s predictive ability for single traits in multi-environment trials (Figure 1k).

**Table 2:** Machine learning (ML) predictive tools validated within a GP framework that can be extended to assist clustering efforts to differentiate between abiotic stress tolerant and susceptible germplasm accessions. These ML algorithms could be trained to distinguish habitat types and local adaptation to heat and drought stresses by looking into *in situ* climate variables and physiological indices from each accession’s geo-referencing (Figure 1b). This is possible because crop wild relatives and landraces have occupied local niches (e.g., hot vs. cold regions) for enough time to be shaped by natural selection. Predicted heat and drought tolerance can further harness other data types (Figure 1e,f) in order to identify and unlock novel sources of heat and drought tolerance (Figure 1g). The ML trained classification may also speed-up the utilization of tolerant variants by genomic-assisted breeding techniques (Figure 1i and Figure 2g). Table is sorted by species name and by ML approach.

ML Approach(es)	Species	Accessions x Genetic Markers	Reference
RF	Barley	911 × 2146 SNP	Heslot et al., 2012 [139]
ANN	Bean	80 × 384 SNP	Rosado et al., 2020 [140] **Ψ**
SVM	Black tea	255 × 1421 DArT SNP	Koech et al., 2020 [141] **Ψ**
RF	Chickpea	315 × 1568 DArT SNP	Roorkiwal et al., 2016 [142] **Ψ**
DT, Bagging, Boosting, RF, ANN	Coffee	245 × 74 AFLP, 58 SSR, 4 RAPD, and 2 primers	Sousa et al., 2021 [143]
RF	Coffee	96 × 38,106 SNP	Ferrão et al., 2019 [144]
SVM	Hybrid Rice	575 × 116,482 SNP	Xu et al., 2018 [145]
ANN	Maize	300 × 55,000 SNP	González-Camacho, et al., 2012 [146]
DL	Maize	148,452 × 19,465 SNP	Khaki & Wang, 2019 [147]
DL	Maize	~300 × ~1000 SNP	Rachmatia et al., 2017 [148]
KNN	Maize	198 × 75 SSR	Maenhout et al., 2007 [149]
MLP, PNN	Maize	~300 × 46,374 SNP	González-Camacho et al., 2016 [150] **Ψ**
RBFNN, ANN	Maize	~300 × 46,374 SNP	González-Camacho et al., 2012 [146] **Ψ**
RF	Maize	240 × 29,619 SNP	Shikha et al., 2017 [151] **Ψ**
RF	Maize	240 × 56,110 SNP	Shikha et al., 2017 [151]
RF, SVM, ANN, Boosting	Maize	391 × 332,178 SNP	Azodi et al., 2019 [152]
SVM	Maize	4,328 × 564,692 SNP	Zhao et al., 2020 [153]
SVM, RF	Maize	113 × 47,458 SNP	Li et al., 2020 [154] **Ψ**
ZAP-RF	Maize	115 × 1635 SNP	Montesinos-López et al., 2021 [155]
DL	Maize	309 × 158,281 SNP	Montesinos-López, et al., 2018 [135]
RF, SVM	Mice	1,884 × 9917 SNP	Neves et al., 2012 [156]
SVM	Pea	105 × 7521 SNP	Annicchiarico et al., 2017 [157]
RF, Boosting, KNN	Perennial ryegrass	86 × 1670 SNP	Grinberg et al., 2016 [158]
RF, GBM, KNN	Perennial ryegrass	86 × 1670 SNP	Grinberg et al., 2016 [158]
Bagging, RF, SVM	Rice	363 × 73,147 SNP	Banerjee et al., 2020 [159] **Ψ**
RF	Rice	110 × 3071 SNP	Onogi et al., 2015 [160]
SVM, Boosting	Simulated Dataset	3226 × 10,031	Ogutu et al., 2011 [161]
DL	Strawberry	1358 × 9908 SNP	Zingaretti et al., 2020 [162]
ANN	Wheat	599 × 1279 SNP	Gianola, et al., 2011 [163]
ANN	Wheat	306 × 1717 SNP	Pérez-Rodríguez et al., 2012 [164]
DL	Wheat	~500 × 15,744 SNP	Crossa et al., 2019 [133]
DL	Wheat	237 × 27,957 SNP	Guo et al., 2020 [165] **Ψ**
DL	Wheat	2000 × 33,709 DArT SNP	Ma et al., 2017 [166]
GBM, RF, SVM	Wheat	254 × 33,516 SNP	Grinberg et al., 2020 [167] **Ψ**
MLP, PNN	Wheat	~300 × 1717 DArT SNP	González-Camacho et al., 2016 [150] **Ψ**
RF	Wheat	254 × 41,371 SNP	Poland et al., 2012 [168]
RF, KNN	Wheat	273 × 5054 SNP	Arruda et al., 2015 [169]
DL, SVM	Wheat	3486 × 2038 SNP	Montesinos-López et al., 2019 [170]

ML tool abbreviations as follows: adaptive boosting (AdaBoost), artificial neural networks (ANN), decision tree (DT), deep learning (DL), extreme gradient boosting (XGBoost), gradient boosting machines (GBM), multilayer perceptron neural network (MLP), probabilistic neural network (PNN), radial basis function neural network (RBFNN), random forest (RF), support vector machines (SVM), and zero altered Poisson random forest (ZAP-RF). ML-coupled genomic prediction initiatives explicitly related to abiotic stress tolerance are marked with **Ψ** under reference.

Nowadays, it is anticipated that ML will overtake current GP models in the near future by incorporating explicit environmental variables and habitat adaptation indices (Figure 1b) as well as estimates of the genome-wide nucleotide diversity (π, expected heterozygosity—*H_e_*, and runs of homozygosity—ROH). Combining trans-disciplinary datasets from different origins and disciplines will ultimately improve the prediction of the phenotypic variance and the genotype × environment interaction, while sidestepping the restrictions of traditional breeding schemes to increase genetic gain for abiotic stress tolerance per unit of time.

Besides the methodological innovations of ML in terms of data merging and optimized classifications, extensions of this type of research are likely to assist hybrid breeding and polyploid utilization [162], both of which take advantage of dominance and over-dominance effects already present in nature, capable of increasing adaptability to abiotic stresses [31,171]. An antecedent in this direction is that GP can predict untested hybrid genotypes [172], even for perennial crops [125,173,174], by genotyping the potential parental lines and phenotyping a few F1 hybrids. Approximate-Bayesian Computation (ABC) analyses within ML [128,175] may in turn speed up polyploid selection.

In short, ML [138,176,177,178,179] and deep learning approaches [133,170,180,181] promise assisting the conservation [102,182,183,184], managing [185,186], prioritization [187,188,189], and introgression [190,191] of crop wild variation from genebanks (Figure 2). ML may be particularly useful in unexplored isolated pockets of diversity, which contain allelic variants otherwise eroded from modern genotypes [192,193]. These predictive tools may additionally accommodate multi-trait [194] multi-environment [195,196] scores within the growing fields of enviromics [197,198,199], green house speed phenotyping [126,200], simulation-based breeding [99,124], hyperspectral imaging (HSI) [201,202,203], and epigenetics [204], allowing for better resolution and pace to detect and use standing adaptation.

Extending these ultimate analytical approaches will help to resolve major basic questions in the field [205] such as to which scale diversifying selection promotes [206] and gene flow precludes [207,208] local adaptation, and whether large haplotype blocks are pervasive in adaptation signatures due to structural variants [209] such as chromosomal inversions [210,211]. Targeting these issues will allow trans-disciplinary research teams to move into more applied matters like (1) how GP and ML can be intermingled to turbocharge genebanks [212] while disentangling the correlated trade-off complexity in proxy traits as part of speed breeding strategies [125,126], and (2) what is the optimum pathway for pan-genomes [213] and epigenetics [214] to unlock the properties of plants for sustainable development [215]. Approaching these enquiries would enable genomics to cut threats of future global food security from climate change by harnessing crop wild diversity and genebank utilization. After all, a key promise of agriculture genomics [216] is to potentiate genebanks through impact-oriented pre-breeding [217] in the face of the current alimentary and climate emergency [218].

## 5. Conclusions

Mining the biodiversity in genebanks [4] and the cryptic diversity in nature [219] is a prerequisite to overcome food shortages due to increasing temperatures and drought. While climate data and genomics can speed up the identification (Figure 1c) of novel sources of tolerance, merging these data types more cohesively into cutting edge analytical frameworks like GP and ML will aid unlocking (Figure 1h) these new variants, so that they can realistically be transferred into the cultivated genepools, and ultimately, the farmers’ fields. In order to start moving toward this goal, genebank research should focus on (1) studying the genetic and ecological mechanisms of adaptation and diversification in crop species, while (2) investigating the genetic potential of wild relatives to cope with abiotic stresses, both by examining historical and novel germplasm materials. The research community must pay close attention to the discovery of locally adapted varieties and landraces (Figure 1d).

Genomic signatures of adaptation to dry and semi-arid habitats (Figure 1b) are useful for germplasm characterization, enhancing the identification of novel sources of variation, future genome-assisted selection, and crop improvement for abiotic stress tolerance. Ultimately, these analytical approaches, coupled with ML, promise improving the accuracy of parent selection to be used in pre-breeding and breeding steps, besides reinforcing the relevance of crops and their wild relatives as systems to address classic questions in the fields of quantitative genetics, evolutionary biology, and ecological genetics.

Looking forward, the discipline of quantitative genetics is merging with the field of ‘big data’ analytics, a ground for ML (Figure 1). An expanded view of complex traits is arising, moving from a polygenic framework to a view in which all genes are liable to affect adaptation to abiotic stresses [220], so that most heritability can be explained by the effects of rare variants [221,222] and their interactions with epigenetic factors [223]. However, looking back toward the bases of crop evolution [23,24] still has the potential to improve agriculture because natural selection has tested more options than humans ever will [35]. We envision that mining the molecular footprint of selection and adaptation in wild crop relatives and early landraces for plant breeding [224] and climate adaptation [125,225] will benefit from bridging the gap between phenotyping (Figure 1a) and genotyping (Figure 1e) in genebanks [226,227], and the more classical and deterministic conceptual models from the fields of quantitative and population genetics.

## 6. Perspectives

In terms of required innovations, it would be desirable to develop high-resolution fitness maps for various types of stresses [228], and to standardize genome-wide polygenetic scores (GPS) [229] by minimizing genomic pseudo-correlation or linkage disequilibrium (LD) [230,231,232], in order to predict novel abiotic stress tolerant sources in concert with other resistances and nutritional quality for the poorest. It is also becoming more essential to assess second-order genomic-context dependent associations (epistatic effects) as a tool to point toward more complex infinitesimal polygenic systems [233] capable of accounting for the missing heritability [58,234,235] in adaptation, while enhancing innovative genomic-assisted selection strategies that do not exclusively rely on MAS and GP, but also on ML. 

Meanwhile, ML would be obsolete to speed up the breeding of resilient crops if heterogeneous datasets are not easily accessible and their maintenance is not well funded in the long-term. Germplasm accessions [36,226], previous and ongoing prioritizations [37,38,188,236], climate-based habitat inferences on the adaptive [47] and yield [126,237] potentials, genomic screenings [238] and rankings [239], field trials in contrasting environments [9,240], and agricultural surveys for farmers (Figure 1l), should all be made public access in order to pivot ML [241,242] breeding technologies [243]. Open access resources [244] and data sharing [245,246] are also essential to target new population-based collections [38] and better characterize the isolated pockets of diversity that still persist in the hidden hills and valleys of mega diverse hotspots. Without platforms for biological discovery [110] and predictors of the future’s uncertainty [247], further pre-breeding would exclusively rely on a narrow diversity without sufficient pre-adaptation to abiotic stresses, hence jeopardizing crop adaptation. As a strategy for feeding the future [4], the identification, conservation, and utilization of novel sources of heat and drought tolerance among crop wild relatives, some of them already kept in existing genebanks [226], will enable diversifying selection in plant breeding by offsetting the domestication winnowing effect on natural genetic variation [247].

## Data Availability

Data Availability concerning the literature included in this review can be found in the citations hereinafter.
